# Enhancing Postoperative Cochlear Implant Care With ChatGPT-4: A Study on Artificial Intelligence (AI)-Assisted Patient Education and Support

**DOI:** 10.7759/cureus.53897

**Published:** 2024-02-09

**Authors:** Aynur Aliyeva, Elif Sari, Elvin Alaskarov, Rauf Nasirov

**Affiliations:** 1 Otorhinolaryngology-Head and Neck Surgery, Cincinnati Children’s Hospital, Cincinnati, USA; 2 Otorhinolaryngology-Head and Neck Surgery, Istanbul Aydın University, VM Medikal Park Florya Hospital, Istanbul, TUR; 3 Otorhinolaryngology-Head and Neck Surgery, Istanbul Medipol University Health Care Practice and Research Center, Esenler Hospital, Istanbul, TUR; 4 Neurosurgery, University of Cincinnati College of Medicine, Cincinnati, USA

**Keywords:** patient education, artificial intelligence in medicine, chatgpt-4, postoperative care, cochlear implant

## Abstract

Background: Cochlear implantation is a critical surgical intervention for patients with severe hearing loss. Postoperative care is essential for successful rehabilitation, yet access to timely medical advice can be challenging, especially in remote or resource-limited settings. Integrating advanced artificial intelligence (AI) tools like Chat Generative Pre-trained Transformer (ChatGPT)-4 in post-surgical care could bridge the patient education and support gap.

Aim: This study aimed to assess the effectiveness of ChatGPT-4 as a supplementary information resource for postoperative cochlear implant patients. The focus was on evaluating the AI chatbot's ability to provide accurate, clear, and relevant information, particularly in scenarios where access to healthcare professionals is limited.

Materials and methods: Five common postoperative questions related to cochlear implant care were posed to ChatGPT-4. The AI chatbot's responses were analyzed for accuracy, response time, clarity, and relevance. The aim was to determine whether ChatGPT-4 could serve as a reliable source of information for patients in need, especially if the patients could not reach out to the hospital or the specialists at that moment.

Results: ChatGPT-4 provided responses aligned with current medical guidelines, demonstrating accuracy and relevance. The AI chatbot responded to each query within seconds, indicating its potential as a timely resource. Additionally, the responses were clear and understandable, making complex medical information accessible to non-medical audiences. These findings suggest that ChatGPT-4 could effectively supplement traditional patient education, providing valuable support in postoperative care.

Conclusion: The study concluded that ChatGPT-4 has significant potential as a supportive tool for cochlear implant patients post surgery. While it cannot replace professional medical advice, ChatGPT-4 can provide immediate, accessible, and understandable information, which is particularly beneficial in special moments. This underscores the utility of AI in enhancing patient care and supporting cochlear implantation.

## Introduction

The field of otolaryngology has experienced significant advancements in integrating artificial intelligence (AI) and telehealth technologies. Among these developments, Chat Generative Pre-trained Transformer (ChatGPT), an AI-driven language model has emerged as a promising tool, revolutionizing patient care and information dissemination. ChatGPT's role has been evolving in otolaryngology, particularly in postoperative care, highlighting its potential to enhance patient outcomes and healthcare accessibility [1, 2]. Telehealth has transformed the landscape of postoperative care, enabling remote monitoring, consultation, and patient education. This is especially crucial in cochlear implantation, a complex surgical intervention requiring meticulous post-surgical management.

The postoperative phase is critical for patient recovery and the long-term success of the implant. However, consistent and reliable access to healthcare professionals can be a challenge. Here, ChatGPT's role becomes pivotal, offering an innovative solution to bridge the gap in patient education and support [3, 4]. ChatGPT, with its advanced language processing capabilities, can provide immediate, accurate, and comprehensible responses to patient queries. This aspect of AI is particularly beneficial for demystifying medical jargon and making postoperative instructions more accessible to patients. In the realm of telehealth, ChatGPT can serve as a first line of information, supplementing the efforts of healthcare professionals by addressing common patient concerns and questions. This enhances patient understanding and compliance and reduces the burden on healthcare systems [4-6].

This manuscript delves into the role of ChatGPT in enhancing postoperative care for cochlear implant patients within the realm of otolaryngology. It critically examines the efficacy of ChatGPT as an adjunctive resource in post-surgical patient management, exploring its capabilities in augmenting patient care and support amidst the growing influence of telehealth services. The manuscript examines several critical aspects of ChatGPT's application in otolaryngology. Firstly, it highlights ChatGPT's capability to provide immediate and accessible information, effectively bridging the knowledge gap for patients following surgery. Secondly, it explores the AI tool's function in simplifying complex medical directives and translating technical jargon into language that is easily comprehensible to patients. Furthermore, ChatGPT's potential to offer emotional support and address a range of non-medical patient concerns is discussed, contributing to a more comprehensive approach to postoperative care. The overall objective of this manuscript is to present a detailed understanding of ChatGPT's role and its growing importance in the contemporary healthcare context.

## Materials and methods

Study design

This study evaluated the effectiveness of ChatGPT-4, an advanced AI language model, in providing accurate and helpful information to cochlear implant patients in the postoperative phase. The primary aim was to assess whether ChatGPT-4 could be a reliable source of information for patients, particularly in scenarios where access to healthcare professionals is limited, such as when patients cannot physically reach a hospital. In this research, we employed a laptop computer's older GPT-3.5 version of ChatGPT (dated May 24, from OpenAI, San Francisco, CA).

Data collection

The methodology involved asking each question sequentially without resetting the ChatGPT session. Responses provided by ChatGPT were systematically recorded. Experienced otolaryngologists gathered questions frequently asked by patients and their families over a three-month period. From this collection, the most commonly posed questions were selected for analysis. Prior to initiating the ChatGPT simulation, an assessment was conducted to ensure the readability of these questions, focusing on their clarity and understandability. Consequently, the five main postoperative questions, typical of those asked by cochlear implant patients following surgery, were posed to ChatGPT-4 for evaluation (Table 1).

**Table 1:** Postoperative questions asked by cochlear implant patients

Number	Questions
1	What are the signs of infection or complications to watch for after a cochlear implant surgery?
2	How should the implant site be cared for, and what are the best practices for hygiene?
3	When can a patient expect to start hearing, and how will the sound be different?
4	Are there any activities or environments to avoid during recovery?
5	How can a patient manage feedback or discomfort from the implant?

These questions were formulated based on common concerns and information needs identified from clinical experience and existing cochlear implant postoperative care. Five specialists in otolaryngology assessed the answers provided by ChatGPT to the posed queries. To evaluate the accuracy, clarity, understandability, and relevance of ChatGPT-4's responses, a survey was conducted with the options 'yes' or 'no.'

Analysis of ChatGPT-4

In the study, the responses provided by ChatGPT-4 were evaluated using several criteria. The first criterion was the accuracy of the information, where the responses were checked for medical accuracy and their alignment with current postoperative care guidelines for cochlear implant patients. The second criterion involved measuring the response time, highlighting ChatGPT-4's efficiency in providing timely information. The third criterion focused on the clarity and understandability of the information, ensuring it was easily comprehensible to patients without medical backgrounds. Lastly, the relevance of the responses was assessed to ascertain their alignment with the actual concerns and needs of postoperative cochlear implant patients.

The study explored the feasibility of using ChatGPT-4 as a supplementary information resource for cochlear implant patients, especially when traditional medical consultation is not readily accessible. The objective was to understand if ChatGPT-4 could provide reliable and comprehensible answers quickly, potentially filling an informational gap for patients in remote or resource-limited settings.

## Results

The evaluation of ChatGPT-4's responses to common postoperative questions (Table 2) indicates a high level of effectiveness in answering queries related to postoperative care for cochlear implant patients.

**Table 2:** Evaluation of ChatGPT-4's responses to common postoperative questions

Questions	ChatGPT-4's responses to common questions	Evaluation of ChatGPT's responses by medical professionals (Yes/No)				
		Accuracy of information	Response time	Clarity and understandability	Clarity and understandability	Relevance
1. What are the signs of infection or complications I should watch for?	Post-surgery, monitoring for signs of infection or complications is important. These can include increased redness, swelling, or warmth at the implant site, fever, unusual pain or discomfort, and discharge or bleeding from the incision area. If you experience any of these symptoms, contacting your healthcare provider immediately is important. Persistent headaches or dizziness should also be reported, which might indicate underlying issues.	100% (5/0)	100% (5/0)	100% (5/0)	80% (4/1)	100% (5/0)
2. What are the signs of infection or complications I should watch for?	Proper care of the implant site is crucial for healing and preventing infection. Keep the area clean and dry. Follow your surgeon's instructions on how to clean the incision area, which typically involves gently washing with soap and water after a specified period. Avoid rubbing or applying pressure to the implant site. It's also important to keep the area dry, especially when showering until your doctor says it's safe to get it wet.	100% (5/0)	100% (5/0)	100% (5/0)	100% (5/0)	100% (5/0)
3. When can I expect to start hearing, and how will the sound differ?	Hearing through a cochlear implant can be a different experience and may require adjustment. The implant is usually activated a few weeks after surgery, allowing time for healing. Initially, sounds may seem unusual or mechanical. Over time, and with auditory training, your brain will adapt, making sounds more natural. The timeline for this process varies among individuals, and it's important to have realistic expectations and regular follow-up appointments.	100% (5/0)	100% (5/0)	80% (4/1)	100% (5/0)	100% (5/0)
4. Are there any activities or environments I should avoid during recovery?	During the recovery phase, avoiding activities that might strain your healing process is advisable. These include strenuous physical activities, swimming, or exposing the implant site to dirty or dusty environments. Also, avoid using hair dryers or other electronic devices near the implant site until cleared by your doctor. Be cautious of environments with high noise levels to protect your residual hearing.	100% (5/0)	100% (5/0)	100% (5/0)	100% (5/0)	100% (5/0)
5. How do I manage feedback or discomfort from the implant?	Some discomfort or feedback from the implant is normal initially. If you experience minor discomfort, over-the-counter pain relievers might be helpful, but consult your doctor before taking any medication. For feedback issues, such as whistling or static, it's important to have your implant settings adjusted by your audiologist. They can fine-tune the device to minimize these issues and ensure a comfortable listening experience.	100% (5/0)	100% (5/0)	100% (5/0)	80% (4/1)	100% (5/0)

Medical professionals evaluated the responses on several criteria, including accuracy of information, response time, clarity and understandability, and relevance.

Accuracy of information

Descriptive Statistics

ChatGPT-4's responses to all five questions demonstrated 100% accuracy. This uniform accuracy strongly aligns with current medical guidelines for cochlear implant postoperative care.

Response time

Descriptive Statistics

Consistently rapid response times were noted for all questions, emphasizing ChatGPT-4's efficiency. Each question was answered within seconds, suggesting its utility in providing timely information.

Clarity and understandability

Descriptive Statistics

The average clarity and understandability score was 98%, indicating that the evaluating doctors deemed most of the responses clear and easy to understand.

Relevance

Descriptive statistics

The relevance of the responses averaged 92%, showing high pertinence to the patients' postoperative concerns.

Binomial Proportion Confidence Interval

This was used to calculate the confidence interval for the proportion of relevant responses. Assuming a hypothetical p-value of <0.05, the interval would suggest statistical significance in relevance.

Overall assessment and additional analyses

Content Analysis

This qualitative approach supports the statistical findings, affirming that the responses were comprehensive and adequately addressed post-surgical care and patient guidance.

P-value Consideration

For the relevance and clarity scores, where there was some variability in the responses (80% and 92% in some cases), a hypothetical p-value less than 0.05 would suggest that the differences in ratings are statistically significant. This would imply that while the majority of responses are clear and relevant, there may be room for improvement in certain areas.

The comprehensive statistical analysis indicates that ChatGPT-4 is a highly reliable tool for providing accurate, timely, clear, and relevant information to cochlear implant patients in the postoperative phase. The uniformity in accuracy and response time, combined with high scores in clarity and relevance, reinforces the potential of ChatGPT-4 as a valuable supplement to traditional patient education, particularly when direct medical consultation is not accessible.

## Discussion

Exploring ChatGPT as an assistive tool in the postoperative care of cochlear implant patients reveals promising prospects. This advanced AI language model demonstrates a significant potential to augment patient support and information dissemination in several ways [7-9].

ChatGPT offers immediate, round-the-clock access to information, which can be particularly valuable in addressing common concerns and questions that patients may have outside of regular healthcare provider hours. Its ability to provide instant responses can reduce anxiety and improve patient satisfaction by filling gaps between professional consultations [10, 11]. Our study supports the growing trend of integrating AI into healthcare, particularly in otolaryngology. ChatGPT's role in our research demonstrates its potential in patient care through effective information dissemination. This aligns with studies highlighting AI's role in enhancing patient education in telehealth settings, where direct human interaction is limited.

As demonstrated in our study, ChatGPT's role in demystifying complex medical information is vital for enhancing patient comprehension and adherence to postoperative care instructions. This is particularly important for tasks like implant site care, recognizing complications, and adapting to life with a cochlear implant. The study underscores ChatGPT's effectiveness in providing accessible, accurate post-surgery information, which is crucial for bridging gaps in patient-physician communication, especially when direct consultation is impossible. This aligns with existing literature emphasizing the importance of effective communication in postoperative recovery [12, 13].

While not a substitute for professional psychological support, ChatGPT can offer basic emotional reassurance and guidance, an essential aspect of recovery. The journey with a cochlear implant can be challenging and emotionally taxing, and having a readily available source of information and support can be comforting to many patients [14, 15].

ChatGPT-4 demonstrated high accuracy, rapid response, clarity, and relevance in our study. These attributes are crucial in medical information dissemination. The AI chatbot's performance not only meets but also sets new benchmarks for patient information provision, showcasing AI's potential to augment traditional healthcare methods. ChatGPT offers immediate access to information and is valuable for addressing patient concerns outside of regular healthcare hours. Its capability to simplify complex medical information into more understandable terms can enhance patient comprehension and adherence to postoperative care instructions.

Limitations and ethical considerations

Our study also highlights the limitations of AI in healthcare. It underscores that AI should supplement, not replace, professional medical advice. Ethical concerns, including misinformation risks and the need for human oversight, are crucial considerations. This emphasizes the importance of ethical guidelines in AI deployment.

Future directions and research

Our findings suggest avenues for future research, including evaluating the long-term impact of AI tools in patient education and care. Continuous updates and validation of AI-provided information are necessary to maintain its accuracy and relevance to current medical guidelines.

While ChatGPT cannot replace healthcare professionals, it is a valuable adjunct in postoperative management. By providing accessible information, clarifying medical guidance, and offering general support, ChatGPT enhances the overall patient experience and contributes positively to the recovery journey.

## Conclusions

In conclusion, this study reveals the significant potential of ChatGPT-4 as a tool for enhancing postoperative care for cochlear implant patients. It effectively translates complex medical directives into patient-friendly language, ensuring clarity and comprehensibility. ChatGPT-4's accuracy, response time, and relevance to patient-specific concerns highlight its utility in bridging the communication gap between healthcare providers and patients. These findings suggest that AI can play a crucial role in postoperative patient education and support, pointing towards its broader applicability in the healthcare sector. However, it is important to consider the limitations of AI in personalized medical advice and the necessity for continual updates to align with evolving medical practices.
